# A Silent Saboteur of Immunotherapy: Antibiotic Use and Its Impact on Immune Checkpoint Inhibitors Efficacy, a Systematic Review and Meta-Analysis of Recent Studies

**DOI:** 10.3390/cancers18050869

**Published:** 2026-03-08

**Authors:** Giuliana Ciappina, Enrica Toscano, Giordana Di Mauro, Tindara Franchina, Francesca Basile, Gianluca Vanni, Gaetano Facchini, Guglielmo Nasti, Vincenzo Quagliariello, Nicola Maurea, Mariapia Marafioti, Antonio Bottari, Oreste Claudio Buonomo, Alessandro Ottaiano, Massimiliano Berretta

**Affiliations:** 1 Department of Medical Sciences, Section of Experimental Medicine, University of Ferrara, 44121 Ferrara, Italy; gciappina@unime.it; 2 Applied Biology and Experimental Medicine, Department of Chemical, Biological, Pharmaceutical and Environmental Sciences, University of Messina, 98166 Messina, Italy; 3 Division of Medical Oncology, “G. Martino” University Hospital, University of Messina, 98124 Messina, Italy; enrica.toscano@polime.it; 4 School of Specialization in Medical Oncology, Department of Human Pathology “G. Barresi”, University of Messina, 98122 Messina, Italy; giordana.dimauro@studenti.unime.it (G.D.M.); francesca.basile2@studenti.unime.it (F.B.); mariapia.marafioti@studenti.unime.it (M.M.); 5 Translational Molecular Medicine and Surgery, Department of Clinical and Experimental Medicine, University of Messina, 98122 Messina, Italy; 6 Department of Human Pathology of Adulthood and Childhood “G. Barresi”, University of Messina, 98122 Messina, Italy; tindara.franchina@unime.it; 7 Breast Unit, Department of Surgical Science, PTV Policlinico Tor Vergata University, 00133 Rome, Italy; gianluca.vanni@uniroma2.it (G.V.); o.buonomo@inwind.it (O.C.B.); 8 Division of Oncology, Santa Maria delle Grazie Hospital, Azienda Sanitaria Locale Napoli 2 Nord, 80078 Pozzuoli, Italy; gaetano.facchini@aslnapoli2nord.it; 9 Division of Innovative Therapies for Abdominal Metastases, Istituto Nazionale Tumori IRCCS Fondazione G. Pascale, 80131 Naples, Italy; g.nasti@istitutotumori.na.it (G.N.); a.ottaiano@istitutotumori.na.it (A.O.); 10 Division of Cardiology, Istituto Nazionale Tumori-IRCCS-Fondazione G. Pascale, 80131 Napoli, Italy; v.quagliariello@istitutotumori.na.it (V.Q.); n.maurea@istitutotumori.na.it (N.M.); 11 Radiology Unit, Department of Biomedical, Dental Science and Morphological and Functional Images, University of Messina, 98124 Messina, Italy; abottari@unime.it; 12 Department of Clinical and Experimental Medicine, University of Messina, 98122 Messina, Italy

**Keywords:** immune checkpoint inhibitors, antibiotic therapy, gut microbiota, meta-analysis, non-small cell lung cancer

## Abstract

Background: Immune checkpoint inhibitors (ICIs) have transformed the management of solid tumors, yet only a subset of patients achieve durable benefit. The gut microbiota is a key modulator of antitumor immunity, and systemic antibiotic therapy (ABT), frequently prescribed—and sometimes overused—in oncology, can profoundly disrupt microbial homeostasis. Observational studies suggest that ABT may impair ICI efficacy, but results remain heterogeneous, warranting an updated synthesis. Methods: We conducted a systematic review and meta-analysis in accordance with the PRISMA 2020 guidelines. PubMed, Scopus, and EMBASE were searched for studies published between 2018 and 2025 evaluating the association between ABT exposure and time-to-event outcomes in patients with solid tumors treated with ICIs. Studies were required to report explicit definition of the ABT exposure window. Random-effects models were considered primary. A sensitivity analysis was performed in non-small cell lung cancer (NSCLC). Results: Fifteen studies encompassing 52,489 patients were included. ABT exposure was associated with significantly worse OS (random-effects HR 1.16, 95% CI 1.03–1.29) and PFS (random-effects HR 1.11, 95% CI 0.95–1.27), indicating an increased risk of death and disease progression compared with no ABT exposure. In the NSCLC sensitivity analysis, ABT was consistently associated with inferior PFS and, when accounting for heterogeneity, with significantly reduced OS, supporting the robustness of the association. Conclusions: ABT administered in temporal proximity to ICIs is associated with clinically meaningful worsening of survival outcomes across solid tumors, consistent with microbiome-mediated impairment of immunotherapy efficacy. These findings support cautious ABT stewardship in patients receiving ICIs and highlight the need for prospective studies integrating microbiome profiling and standardized ABT exposure assessment.

## Simple Summary

Immune checkpoint inhibitors (ICIs) have improved outcomes in many solid tumors, but durable responses occur in only a minority of patients. Given the role of the gut microbiota in shaping antitumor immunity, systemic antibiotic therapy (ABT) may impair the effectiveness of immune checkpoint inhibitors (ICIs) by inducing gut dysbiosis. This PRISMA 2020-compliant systematic review and meta-analysis searched PubMed, Scopus, and EMBASE for studies published from 2018 to 2025 that evaluated ABT exposure (with clearly defined timing windows) and survival outcomes in ICI-treated solid tumors. Fifteen studies including 52,489 patients were analyzed primarily using random-effects models. ABT exposure was associated with worse overall survival (HR 1.16, 95% CI 1.03–1.29) and a trend toward worse progression-free survival (HR 1.11, 95% CI 0.95–1.27). In a sensitivity analysis of non-small cell lung cancer, ABT was consistently linked to inferior outcomes, supporting the association. These findings argue for careful antibiotic stewardship during ICI therapy and for prospective studies incorporating microbiome profiling.

## 1. Introduction

Immune checkpoint inhibitors (ICIs) have revolutionized the treatment of several solid tumors and now constitute a central component of contemporary oncologic therapy [1]. Nevertheless, durable clinical benefit is achieved in only a subset of patients, highlighting the urgent need to identify host- and treatment-related factors that modulate immunotherapy efficacy and survival outcomes [2,3,4]. In this context, the gut microbiota has emerged as a key regulator of systemic immune homeostasis and antitumor immune responses. A growing body of preclinical and clinical evidence demonstrates that both the diversity and taxonomic composition of the intestinal microbiome influence responsiveness to ICIs through multiple mechanisms, including enhanced antigen presentation, priming and expansion of effector T-cell populations, and modulation of inflammatory and metabolic signaling pathways [5,6]. Conversely, microbiota disruption, commonly referred to as dysbiosis, has been associated with impaired immune surveillance and attenuated efficacy of immune-based therapies [7]. Antibiotics (ABTs) represent one of the most frequently prescribed classes of medications in oncology, used for the treatment and prophylaxis of infections across all phases of cancer care. Their use is often unavoidable in immunocompromised patients; however, ABTs are also recognized to be variably prescribed and, in some settings, potentially overutilized, including empiric or prolonged courses without microbiological confirmation [8,9]. Given their rapid and profound impact on gut microbial ecosystems, ABTs can markedly reduce microbial diversity, eradicate beneficial commensal species, and induce persistent alterations in microbiota composition and function [10]. Mechanistically, ABT-induced dysbiosis can disrupt microbial-derived immunostimulatory signals that support dendritic-cell maturation and antigen presentation, ultimately impairing the priming and maintenance of effector CD8^+^ T-cell responses. In addition, depletion of commensal taxa associated with favorable ICI responsiveness (e.g., Akkermansia, Bifidobacterium, Faecalibacterium) and reduced production of microbial metabolites involved in immune regulation may weaken interferon-driven and T-cell-mediated antitumor immunity [11,12]. These effects raise biologically plausible concerns regarding their capacity to impair antitumor immunity and compromise the clinical efficacy of ICIs. Several observational studies have reported an association between ABT exposure and inferior clinical outcomes in patients receiving ICIs, including reduced progression-free survival (PFS) and overall survival (OS), particularly in melanoma and lung cancer cohorts [13]. However, heterogeneity across studies in terms of tumor types, ABT classes, timing of exposure, and clinical endpoints has contributed to ongoing debate regarding the consistency and magnitude of this effect. A comprehensive systematic review and meta-analysis published in 2023 by Crespin et al. evaluated over 100 studies encompassing more than 41,000 patients and demonstrated that ABT use around ICI initiation was consistently associated with significantly worse OS, PFS, objective response rate (ORR), and progressive disease rate, with the strongest detrimental effect observed when ABTs were administered shortly before or after the start of immunotherapy [14]. While this work provided robust evidence supporting a negative association between ABT exposure and ICIS outcomes, the rapidly evolving landscape of immuno-oncology and the continuous publication of new studies warrant an updated synthesis of the evidence. Accordingly, the aim of the present study was to perform an updated systematic review and meta-analysis assessing the impact of ABT exposure on time-to-outcomes in patients with solid tumors treated with ICIs, incorporating studies published between 2018 and 2025. By integrating the most recent data, this analysis seeks to refine current estimates, evaluate temporal and tumor-specific effects, and provide clinically relevant evidence to guide ABT stewardship in the era of cancer immunotherapy.

## 2. Materials and Methods

This study presents a systematic review and meta-analysis aimed at evaluating the association between ABT use and time-to-event outcomes in patients receiving ICIs. The review was conducted following the 2020 Preferred Reporting Items for Systematic Reviews and Meta-Analyses (PRISMA) guidelines [15] and according to a pre-defined protocol, which was prospectively registered in the PROSPERO database (hosted by the National Institute for Health Research, London, UK) under registration number CRD420251269646. The completed PRISMA 2020 checklist is available as Supplementary File S1.

### 2.1. Search Strategy and Selection Criteria

A comprehensive literature search was performed in PubMed, Scopus, and EMBASE to identify relevant studies evaluating the impact of ABT exposure on clinical outcomes in cancer patients treated with ICIs. The search strategy combined terms related to immunotherapy, ABTs, and survival outcomes, and was constructed as follows: (“Immunotherapy”[Mesh] OR immunotherapy OR immune checkpoint inhibitors OR ICIs OR ICI OR nivolumab OR pembrolizumab OR atezolizumab OR ipilimumab OR durvalumab OR dostarlimab) AND (“Anti-Bacterial Agents”[Mesh] OR antibiotics OR “antibiotic type” OR “beta-lactams” OR “fluoroquinolones” OR “macrolides” OR “cephalosporins” OR “carbapenems” OR “penicillins”) AND (“Neoplasms”[Mesh] OR cancer OR tumor OR malignancy OR oncology) AND (“Treatment Outcome”[Mesh] OR survival OR progression OR “treatment efficacy”). The search covered publications from January 2018 to September 2025. A manual search was also conducted to identify additional studies and ensure contextual relevance. Six investigators, divided into two independent teams, screened titles, abstracts, and full texts to select eligible studies. The inclusion criteria were as follows: retrospective or prospective clinical studies; studies evaluating the prognostic impact of ABT use in patients with solid tumors receiving ICIs, either as monotherapy or in combination; studies reporting OS or PFS with hazard ratios (HRs) and 95% confidence intervals (CIs); explicit reporting of the ABT exposure window, with hazard ratios (HRs extracted for the exposure window closest to ICIs administration, see also Supplementary File S2). Exclusion criteria included: animal studies; reviews, commentaries, letters, or conference abstracts; case reports or case series; studies unrelated to the research question; studies with incomplete data or in which HRs and 95% CIs could not be extracted. The PRISMA flowchart illustrating the study selection process is reported in Supplementary Figure S1.

### 2.2. Data Extraction

From each included study, the following data were extracted: first author, year of publication, study design, sample size, median age, gender distribution, cancer type, immunotherapy regimen (including ICI type and line of therapy), ABT type, number of ABT users and non-users, timing of ABT administration, and time-to-outcomes with HR and 95%CIs. We systematically extracted the maximally adjusted hazard ratio reported in each study in order to minimize the potential impact of confounding effects.

### 2.3. Primary Objective

The primary objective of this systematic review and meta-analysis was to assess the impact of ABT exposure on time-to-event outcomes, specifically OS and PFS, in patients with solid tumors treated with ICIs.

### 2.4. Quality Assessment

Two independent reviewer teams critically evaluated the methodological quality and risk of bias of the included studies. Quality appraisal was performed using the Methodological Index for Non-Randomized Studies (MINORS) [16] and the Newcastle–Ottawa Scale (NOS) [17]. Any discrepancies between the two teams were resolved through consensus discussions.

### 2.5. Statistical Methods

A meta-analytic approach was applied to evaluate the association between ABT exposure and clinical outcomes in patients with solid tumors receiving ICIs. The primary effect measure was the HR for OS and PFS, comparing patients exposed to ABT with those not receiving ABTs. Pooled estimates were calculated using both fixed-effect and random-effects models, with the latter implemented according to the DerSimonian and Laird method [18]. The fixed-effect model assumes a common underlying treatment effect across studies, whereas the random-effects model allows for variability in true effect sizes due to differences in study populations, tumor types, ABT exposure definitions, and clinical settings. As recommended in the presence of clinical and methodological heterogeneity, results from the random-effects model were considered primary. Summary HRs were reported with corresponding 95% CIs and visually summarized using Forest plots, displaying individual study estimates alongside the overall pooled effect. An HR equal to 1.0 indicates no difference in the risk of progression or death between ABT-exposed and unexposed patients, while HR values greater than 1.0 reflect inferior outcomes associated with ABT use. Statistical heterogeneity was quantified using Cochran’s Q test and the I^2^ statistic, which describes the percentage of total variability attributable to between-study heterogeneity rather than random error [19]. I^2^ values were interpreted using conventional thresholds: <25% (low), 25–50% (moderate), 50–75% (substantial), and >75% (considerable heterogeneity). Negative I^2^ values were set to zero, in line with standard practice. The presence of substantial heterogeneity prompted additional exploratory analyses. Sensitivity analyses were intentionally limited to patients with non-small cell lung cancer (NSCLC), which represented the largest and most homogeneous body of evidence. This restriction was implemented to evaluate the stability of pooled estimates within a single tumor type supported by an adequate number of studies. Sensitivity analyses were not performed for other cancer histologies due to insufficient study numbers and excessive data fragmentation, which precluded meaningful and reliable secondary analyses. Potential reporting bias was assessed through the visual examination of funnel plots plotting the effect sizes against their standard errors, as recommended for meta-analyses including a sufficient number of studies [20]. Funnel plot asymmetry was further evaluated using Egger’s linear regression test and Begg’s rank correlation test to detect small-study effects and selective publication. All statistical analyses were conducted using MedCalc Statistical Software version 19.6 (MedCalc Software Ltd., Ostend, Belgium), with supplementary data handling performed in Microsoft Excel^®^ for Windows version 2302 (Microsoft Corp., Redmond, WA, USA).

## 3. Results

### 3.1. Characteristics of the Studies

In total, 1680 records were identified through database searching; after the removal of duplicates, 1534 records were screened at the title/abstract level and 1402 were excluded. One hundred and thirty-two full-text articles were assessed for eligibility, and 117 were excluded, mainly because they did not report time-to-event outcomes as hazard ratios. A total of 15 clinical studies met the predefined inclusion criteria and were included in the quantitative synthesis (Table 1), comprising 52,489 patients treated with ICIs [21,22,23,24,25,26,27,28,29,30,31,32,33,34,35]. The studies were published between 2018 and 2025 and encompassed heterogeneous methodological designs, including retrospective analyses (*n* = 10), prospective studies (*n* = 3), and post hoc analyses of clinical trials (*n* = 2). Two publications reported outcomes from independent cohorts within the same study and were therefore analyzed and described as separate datasets. The size of individual cohorts varied substantially, ranging from 66 patients to 41,529 patients, reflecting marked differences in study scale and data sources. Across studies, ICIs included anti-PD-1, anti-PD-L1, and anti-CTLA-4 monoclonal antibodies. Treatment strategies were heterogeneous: eight studies evaluated ICIs in monotherapy, whereas nine studies assessed ICIs administered in association with other anticancer treatments, including chemotherapy, targeted therapies, tyrosine kinase inhibitors, radiotherapy, radioembolization, or monoclonal antibodies. The proportion of ICI-treated patients exposed to systemic ABT showed wide variability, ranging from 13.8% to 52% across cohorts. The ABT-exposure percentages reported across the included studies corresponded to approximately 16,300 antibiotic-exposed patients versus 36,600 non-exposed patients overall. Higher rates of ABT exposure were generally observed in studies evaluating combination regimens, particularly those including chemotherapy or targeted therapies, while lower percentages were more frequently reported in monotherapy cohorts. Only studies reporting time-to-outcome analyses as HRs with corresponding CIs were selected. OS was available across all included studies, whereas PFS was reported in the majority of cohorts, with a limited number of studies providing OS data only. Collectively, the included studies represent a broad and contemporary evidence base, encompassing diverse clinical settings and therapeutic strategies, thereby providing a robust framework for subsequent pooled and subgroup analyses examining the impact of ABT on outcomes in patients treated with ICIs. The methodological quality of the included studies was generally high (Supplementary Table S1). MINORS scores for non-randomized studies ranged from 12 to 20, indicating acceptable to excellent methodological rigor. NOS scores for retrospective and prospective cohorts ranged from 5 to 9, reflecting moderate to high quality in terms of selection, comparability, and outcome assessment.

### 3.2. Baseline Clinical Characteristics of the Included Patient Populations

Baseline demographic and clinical characteristics of patients across the included studies are summarized in Table 2. Overall, the analyzed populations were predominantly male, with the proportion of male patients ranging from 44% to 85.7% across individual cohorts. Median age at study entry was consistently within the sixth to seventh decade of life, varying from 60 to 70 years in studies reporting this parameter, while age data were not available in a limited number of cohorts. The spectrum of tumor types reflected the clinical indications for ICI use in routine practice. NSCLC was the most frequently represented malignancy, either as a single disease entity or within mixed-tumor cohorts. Other tumor types included renal cell carcinoma (RCC), urothelial carcinoma (UC), malignant melanoma (MM), hepatocellular carcinoma (HCC), colorectal cancer (CRC), gastric cancer, sarcoma, and gastrointestinal stromal tumors (GIST). Several studies enrolled patients with multiple tumor histologies, underscoring the pan-tumor application of ICIs in real-world and trial-based settings. With respect to treatment line, ICIs were administered across different phases of the therapeutic trajectory. Most studies included patients treated in any line of therapy, whereas others focused specifically on first-line or second or later lines. Notably, a subset of large and more recent cohorts preferentially evaluated ICI use in the first-line setting, particularly in NSCLC and melanoma populations.

### 3.3. Temporal Window and Qualitative Characteristics of ABT Exposure

As a prerequisite for study selection, particular attention was paid to verifying whether the temporal window of ABT administration relative to ICI initiation was explicitly reported, in order to ensure biological and clinical interpretability of the association analyses. Table 3 summarizes the timing and qualitative characteristics of ABT across the included studies. Overall, there was substantial heterogeneity in the definition of ABT exposure windows, although most studies focused on ABT use occurring in close temporal proximity to ICI initiation. The most commonly adopted window encompassed 30–60 days prior to ICI start, either alone or combined with ABT exposure during early ICI treatment. Several studies extended the exposure window to include concurrent ABT during ICI therapy, typically within the first weeks or months of treatment, reflecting real-world prescribing patterns in advanced cancer patients. A smaller number of studies employed broader temporal windows, ranging from 2–3 months before ICI initiation to 1–3 months after treatment start, and in one large retrospective cohort, ABT exposure was assessed up to more than one year prior to ICI initiation. While these extended windows captured long-term microbiome-disrupting effects, they introduced greater biological heterogeneity in terms of the potential reversibility of ABT-induced dysbiosis. Regarding the type of ABTs, when reported, ABT exposure predominantly involved broad-spectrum agents, with recurrent representation of beta-lactams and fluoroquinolones, followed by macrolides, tetracyclines, sulfonamides, aminoglycosides, carbapenems, and glycopeptides. However, in a considerable proportion of cohorts, particularly large registry-based or post hoc trial analyses, the specific classes of ABTs administered were not reported, limiting finer-grained evaluations of class-specific effects.

### 3.4. Pooled Effect of Systemic ABT on Time-to-Outcome in ICIS-Treated Patients

**Figure 1:**
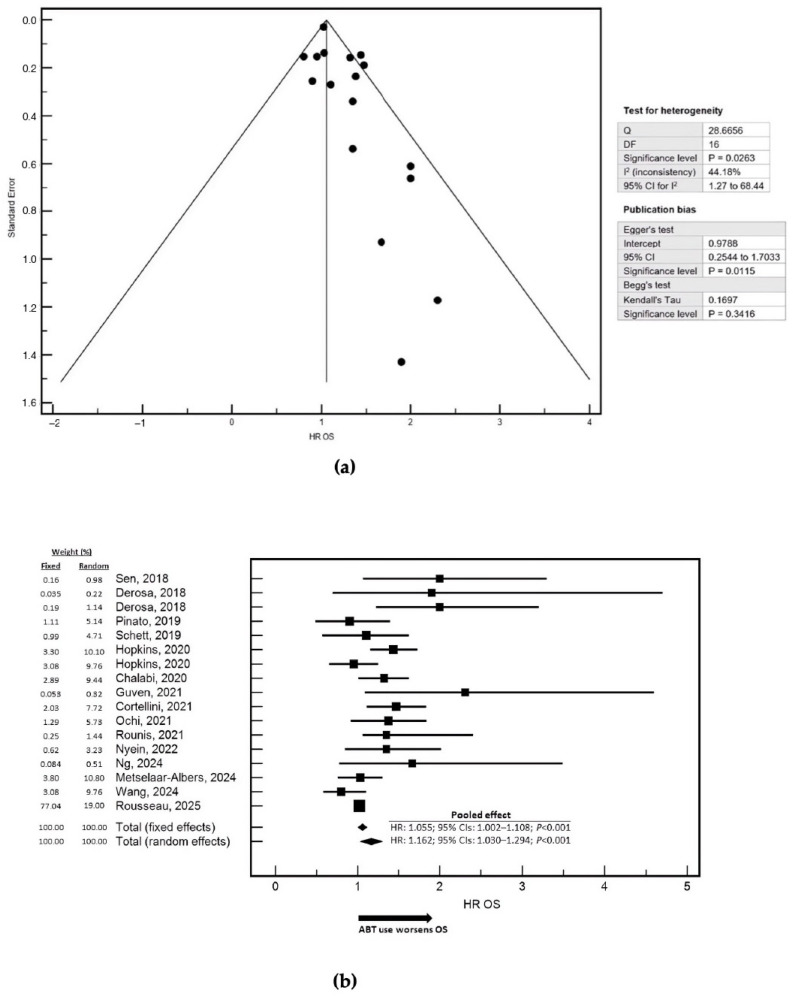
Pooled analysis of the association between systemic ABT and OS in patients treated with immune checkpoint inhibitors [21,22,23,24,25,26,27,28,29,30,31,32,33,34,35]. (**a**) shows the funnel plot assessing potential small-study effects and publication bias for OS. The standard error is plotted on the Y-axis and the HR for OS on the X-axis. To the right of the funnel plot, the results of the heterogeneity and publication bias tests are reported. Between-study heterogeneity was statistically significant, as indicated by Cochran’s Q test (Q = 28.67, degrees of freedom = 16, *p* = 0.0263). The I^2^ statistic was 44.18% (95% CI: 1.27–68.44%), suggesting a moderate level of inconsistency across studies. Assessment of publication bias showed a significant Egger’s test intercept (0.9788; 95% CI: 0.2544–1.7033; *p* = 0.0115), indicating possible small-study effects, whereas Begg’s test based on Kendall’s tau did not demonstrate significant asymmetry (τ = 0.1697; *p* = 0.3416). (**b**) presents the forest plot of the pooled hazard ratios for OS comparing patients receiving systemic antibiotic therapy (ABT) with those not exposed to ABT. Pooled estimates are displayed using both fixed-effects and random-effects models. In the fixed-effects model, ABT exposure was associated with a statistically significant increase in the risk of death (HR = 1.055; 95% CI: 1.002–1.108; *p* < 0.001). This association remained significant and of greater magnitude in the random-effects model (HR = 1.162; 95% CI: 1.030–1.294; *p* < 0.001). The analysis included a total of 52,892 patients. An arrow on the x-axis indicates the direction of effect, showing that ABT use is associated with worse OS relative to no ABT exposure. Study weights, based on sample size and estimate precision, reflect the relative contribution of each study to the pooled estimate.

**Figure 2:**
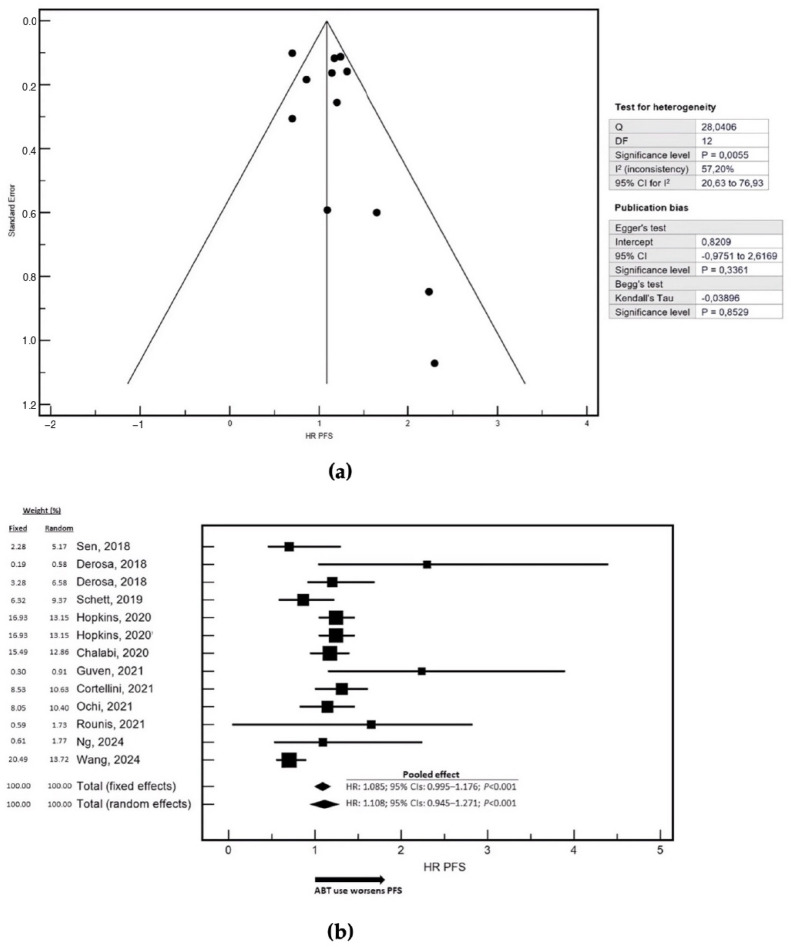
Pooled analysis of the association between systemic antibiotic therapy (ABT) and progression-free survival (PFS) in patients treated with immune checkpoint inhibitors [21,22,24,25,26,27,28,29,30,32,34]. (**a**) shows the funnel plot assessing potential small-study effects and publication bias for PFS. The standard error is plotted on the Y-axis and the hazard ratio (HR) for PFS on the X-axis. To the right of the funnel plot, the results of the heterogeneity and publication bias tests are reported. Between-study heterogeneity was statistically significant, as indicated by Cochran’s Q test (Q = 28.04, degrees of freedom = 12, *p* = 0.0055). The I^2^ statistic was 57.20% (95% CI: 20.63–76.93%), suggesting a moderate-to-high level of inconsistency across studies. Assessment of publication bias showed no significant Egger’s test intercept (0.8209; 95% CI: –0.9751–2.6169; *p* = 0.3361), and Begg’s test based on Kendall’s tau did not indicate asymmetry (τ = –0.03896; *p* = 0.8529). (**b**) presents the forest plot of the pooled HRs for PFS from the meta-analysis, comparing patients receiving systemic ABT with those not exposed. Pooled estimates are displayed using both fixed-effects and random-effects models. In the fixed-effects model, ABT exposure showed a trend toward increased risk of progression (HR = 1.085; 95% CI: 0.995–1.176; *p* < 0.001). Similarly, the random-effects model produced a comparable estimate (HR = 1.108; 95% CI: 0.945–1.271; *p* < 0.001). Data were pooled from a total of 6377 patients. An arrow on the X-axis indicates the direction of effect, suggesting that ABT use may be associated with worse PFS relative to no ABT exposure. Study weights, based on sample size and estimate precision, reflect the relative contribution of each study to the pooled estimate.

We next evaluated the pooled effect of ABT on OS and PFS in patients treated with ICIs. The meta-analysis of OS included 52,892 patients (Figure 1a,b). The selected maximally adjusted HRs and corresponding 95% CIs for OS and PFS are reported in Supplementary File S3. ABT exposure was consistently associated with inferior OS compared with no use. In the fixed-effects model, ABT was associated with a modest but statistically significant increase in the risk of death (HR = 1.055; 95% CI: 1.002–1.108). However, a moderate degree of between-study heterogeneity was observed; therefore, the random-effects model, which provides a more conservative and clinically credible estimate in the presence of heterogeneit, was considered more appropriate for interpretation. Under the random-effects model, ABT exposure was associated with a stronger and statistically significant detrimental effect on OS (HR = 1.162; 95% CI: 1.030–1.294). From a clinical perspective, this translates into an approximately 16% increase in the risk of death for patients receiving ABT compared with those not exposed to ABTs during the defined peri-ICI window. The pooled analysis of PFS was based on 6377 patients (Figure 2a,b). ABT exposure was again associated with worse outcomes. In the fixed-effects model, ABT showed a trend toward increased risk of disease progression (HR = 1.085; 95% CI: 0.995–1.176). Given the presence of moderate-to-high heterogeneity across studies, greater interpretative weight was attributed to the random-effects estimate. In this model, ABT exposure was associated with an increased risk of progression (HR = 1.108; 95% CI: 0.945–1.271). Overall, these findings indicate that ABT use is associated with an approximately 11% increase in the risk of disease progression relative to no ABT exposure in ICI-treated patients. Because three studies specifically reported OS data in patients treated with ICIs in combination with chemotherapy, a separate pooled analysis was conducted for this subgroup (Supplementary Figure S2a,b). No relevant between-study heterogeneity was detected, and fixed- and random-effects models yielded identical estimates. In this setting, ABT exposure was associated with a statistically significant increase in mortality risk (HR = 1.120; 95% CI: 1.080–1.160), corresponding to an approximately 12% higher risk of death compared with patients not receiving ABT. A pooled analysis of PFS in the ICI plus chemotherapy subgroup was not performed, as only a single study reported HR data for this endpoint. A histotype-specific sensitivity analysis evaluating the effect of ABT was conducted in NSCLC (Supplementary File S4).

## 4. Discussion

In this meta-analysis, we provide an updated and biologically grounded evaluation of the impact of ABT on survival outcomes in patients treated with ICIs. By restricting inclusion to contemporary studies explicitly reporting the temporal window of ABT exposure and by prioritizing ABT use closest to ICI initiation, we aimed to maximize both clinical relevance and mechanistic plausibility. Our findings demonstrate that ABT exposure is consistently associated with inferior time-to-outcome endpoints, including both OS and PFS, across a broad spectrum of solid tumors and treatment settings. At the pooled level, ABT exposure was associated with a 16% increased risk of death and an 11% increased risk of disease progression in ICI-treated patients. In the presence of moderate to moderate-to-high heterogeneity for OS and PFS, respectively, greater interpretative weight was given to random-effects models, which are methodologically more appropriate when effect sizes vary across studies due to clinical and biological diversity. The consistency of directionality across analyses further strengthens the robustness of our conclusions. Furthermore, a relevant extension of our work is the subgroup analysis focusing on patients treated with ICIs in combination with chemotherapy. In this setting, ABT exposure was associated with a 12% higher risk of death, with no detectable between-study heterogeneity. This finding suggests that the negative impact of ABTs is not mitigated by the addition of cytotoxic chemotherapy and may even persist in more intensive treatment regimens. A sensitivity analysis was conducted in NSCLC, given the robustness and volume of available evidence in this tumor type (Supplementary File S4). These findings are consistent with the overall results of the meta-analysis and indicate that, within NSCLC, ABT exposure is consistently associated with inferior progression-related outcomes and, when accounting for between-study heterogeneity, with significantly reduced OS, supporting a clinically meaningful negative impact of ABT on immunotherapy efficacy in this tumor type. Interestingly, our results are directionally concordant with, but quantitatively more conservative than, those reported in a large and comprehensive prior meta-analysis by Crespin et al. [14], which showed substantially higher HRs for both OS and PFS. Several factors likely explain this discrepancy. First, the earlier studies included a much broader temporal range of ABT exposure, extending far beyond ICI initiation, which may have amplified effect sizes by capturing more profound or prolonged microbiome disruption. Second, our deliberate exclusion of studies lacking explicit ABT timing and our a priori selection of exposure windows closest to ICI administration reduced heterogeneity and potential misclassification bias, at the cost of attenuating pooled estimates. In this respect, our findings may more accurately reflect the real-world magnitude of risk attributable to ABT in contemporary ICI-treated populations. From a biological standpoint, the observed associations are strongly supported by mechanistic evidence linking the gut microbiota to antitumor immune responses [36]. ABTs, particularly broad-spectrum agents such as beta-lactams and fluoroquinolones, which predominated in the included studies, are known to induce gut dysbiosis, reduce microbial diversity, and deplete commensal taxa involved in antigen presentation, T-cell priming, and interferon signaling [37,38]. The stronger effects seen when ABTs are administered shortly before or during ICI therapy further support a causal, time-dependent interaction between microbiome integrity and immunotherapy efficacy. An apparent contrast to previous and our findings emerged from the recent randomized phase III study by Riaz et al. [39], which comprehensively interrogated tumor genomic, immune, and microbiome features in patients with locally advanced head and neck cancer treated with avelumab plus chemoradiotherapy. In that study, resistance to chemo-immunotherapy was associated not with prior systemic ABT exposure per se, but with the presence of intratumoral bacteria, coupled with a myeloid- and neutrophil-enriched tumor microenvironment, elevated systemic neutrophil-to-lymphocyte ratios, and suppressed adaptive immunity. Importantly, this work highlights a context-dependent role of the microbiome, where localized intratumoral bacterial ecosystems, rather than ABT-induced gut dysbiosis, appear to drive immune dysfunction and impaired response to PD-L1 blockade in a highly specific disease setting. These observations are not necessarily contradictory but instead underscore the spatial, functional, and biological heterogeneity of host–microbiome–tumor interactions [40,41]. Our meta-analysis predominantly captures the systemic consequences of ABT exposure on the gut microbiota and its downstream effects on immune priming and effector function, particularly in metastatic settings and across multiple tumor types. In contrast, the Riaz study focused on a locoregional disease context treated with definitive chemoradiotherapy, where intratumoral bacterial colonization and inflammation-driven myeloid programs may exert dominant, and potentially independent, immunosuppressive effects. Notably, chemotherapy and radiotherapy themselves can profoundly reshape both local microbial niches and immune cell composition [42,43], further differentiating this scenario from ICI monotherapy or systemic combination regimens. Taken together, these data may suggest that the impact of bacteria on immunotherapy efficacy is not unidirectional but instead depends on microbial compartmentalization (gut versus tumor), immune contexture, treatment backbone, and disease stage. While systemic ABTs may impair ICI efficacy by disrupting beneficial gut-derived immune signaling, intratumoral bacteria may promote resistance through chronic innate immune activation and neutrophil-dominated suppression. This conceptual framework reinforces the need for future studies to disentangle systemic versus intratumoral microbiome effects, integrate ABT exposure with spatial microbiome profiling, and tailor microbiome-modulating strategies to specific therapeutic contexts rather than assuming uniform benefit or harm. Our study has several limitations that warrant careful consideration. First, most included studies were retrospective in nature, making them susceptible to confounding by indication, as patients receiving ABTs may have had worse baseline clinical conditions, infections, or more aggressive disease. Although the consistency of findings across large cohorts and treatment settings mitigates this concern, residual confounding cannot be fully excluded. Second, heterogeneity in ABT definitions, exposure windows, and reporting of ABT classes limited our ability to perform detailed class-specific or dose-dependent analyses. In particular, the absence of detailed information on antibiotic dosing in the included studies—together with limited reporting on treatment duration, route of administration, and cumulative exposure—may plausibly influence both the magnitude and persistence of microbiota disruption and, consequently, clinical outcomes during ICI therapy. This lack of granularity precludes reliable data extraction and prevents dose–response or intensity-stratified analyses within our meta-analysis. Moreover, the insufficient reporting of antibiotic dosing across primary studies limits our ability to explore dose-dependent effects and may contribute to residual heterogeneity. Third, and most importantly, we were unable to systematically assess treatment response endpoints, such as ORR or disease control rate (DCR). This was due to the limited availability, heterogeneity, and non-standardized reporting of response data across studies. As a result, our conclusions are confined to time-to-event outcomes and cannot directly address whether ABT primarily affects initial tumor response, durability of benefit, or both.

## 5. Conclusions

This meta-analysis highlights antibiotic exposure as a clinically relevant negative modifier of both efficacy and immune-related toxicity in patients treated with ICIs. These findings underscore the importance of the early identification of patients exposed to antibiotics, who may represent a biologically distinct subgroup with impaired immune responsiveness. Prospective strategies aimed at restoring host–microbiome–immune interactions, including the use of prebiotics, probiotics, and immuno-nutritional interventions, warrant further investigation. Such approaches may help mitigate the detrimental effects of ABT-induced dysbiosis and optimize the therapeutic index of immunotherapy. Well-designed prospective and biomarker-driven studies are needed to define patient selection, timing, and the most effective microbiome-modulating strategies. Prospective and registry-based studies should incorporate granular and standardized antibiotic exposure metrics (including dose, duration, class, and timing relative to ICI initiation) to enable more informative analyses and clarify whether a dose–response relationship exists.

## Figures and Tables

**Table 1:** Characteristics of the selected clinical studies.

Author	Year	Study Design	Patients’ Number	ICI Treatment			% of ICI-Treated Pts Receiving ABT	Time-to-Outcome *
				Type of ICI	Monotherapy (M)/ Association (A)	Type of Non-ICI Associated Treatments		
Sen et al. [21]	2018	Post hoc analysis	172	Anti-CTLA4; anti-PD1	A	RT; TT	52	OS, PFS
Derosa et al. ** [22]	2018	Retrospective	121	Anti-CTLA-4; anti-PD1; anti-PD-L1	A	Bevacizumab	31	OS, PFS
Derosa et al. ** [22]	2018	Retrospective	239	Anti-CTLA-4; anti-PD1; anti-PD-L1	A	None	48	OS, PFS
Pinato et al. [23]	2019	Prospective	196	Anti-PD1; anti-PD-L1	A	None	50	OS
Schett et al. [24]	2020	Retrospective	218	Anti-PD1; anti-PD-L1	M	None	15.1	OS, PFS
Hopkins et al. (IMVigor210) ** [25]	2020	Post hoc analysis	429	Atezolizumab	M	None	26	OS, PFS
Hopkins et al. (IMVigor211) ** [25]	2020	Post hoc analysis	931	Atezolizumab	M	None	26	OS, PFS
Chalabi et al. [26]	2020	Retrospective	1512	Atezolizumab	M	None	22	OS, PFS
Guven et al. [27]	2021	Retrospective	93	Nivolumab	M	None	33	OS, PFS
Cortellini et al. [28]	2021	Prospective	1545	Pembrolizumab	M	None	13.8	OS, PFS
Ochi et al. [29]	2021	Retrospective	531	Anti-PD1; anti-PD-L1	M	None	19	OS, PFS
Rounis et al. [30]	2021	Prospective	66	Anti-PD1; anti-PD-L1	M	None	51.5	OS, PFS
Nyein et al. [31]	2022	Retrospective	256	Anti-CTLA-4; anti-PD1; anti-PD-L1	A	CT; TT	18	OS
Ng et al. [32]	2024	Retrospective	168	NR	A	mAb; TKIs; RE	36.9	OS, PFS
Metselaar-Albers et al. [33]	2024	Retrospective	4534	Anti-CTLA-4; anti-PD1; anti-PD-L1	A	CT	29	OS
Wang et al. [34]	2024	Retrospective	352	Anti-PD1	A	Lenvatinib; regorafenib; sorafenib; others	33.8	OS, PFS
Rousseau et al. [35]	2025	Retrospective	41,529	Pembrolizumab	A	CT	32.1	OS

* This information refers to the reporting of hazard ratios (HRs) with the corresponding confidence intervals (CIs). ** The data refer to two independent cohorts, for which outcomes are reported within the same study. ABT: systemic antibiotic therapy; Anti-CTLA-4: Anti-Cytotoxic T-Lymphocyte-Associated protein 4 monoclonal antibodies; Anti-PD-1: Anti-Programmed cell Death protein 1 monoclonal antibodies; Anti-PD-L1: Anti-Programmed cell Death Ligand 1 monoclonal antibodies; CT: Chemotherapy; ICI: Immune Checkpoint Inhibitor; mAb: Monoclonal Antibody; NR: Not Reported; OS: Overall Survival; PFS: Progression-Free Survival; RE: Radioembolization; RT: Radiotherapy; TKIs: Tyrosine Kinase Inhibitors; TT: Targeted Therapy.

**Table 2:** Baseline clinical characteristics of patients across the included studies.

Author	Year	Percent of Male (%)	Median Age (Years)	Cancer Type	Treatment Line	Stage of Disease
Sen et al. [21]	2018	51	60	RCC, NSCLC, MM, sarcoma, GIST	Second or later	Advanced
Derosa et al. * [22]	2018	66	61	RCC	All	Advanced
Derosa et al. * [22]	2018	49	66	NSCLC	All	Advanced
Pinato et al. [23]	2019	70	68	MM, NSCLC, others	All	84% advanced, 16% localized
Schett et al. [24]	2019	60	63	NSCLC	All	Advanced
Hopkins et al. (IMVigor210) * [25]	2020	78	NR	UC	All	Advanced
Hopkins et al. (IMVigor211) * [25]	2020	76	67	UC	Second	Advanced
Chalabi et al. [26]	2020	62.3	NR	NSCLC	Second or later	Advanced
Guven et al. [27]	2021	76.3	61	RCC	Second or later	Advanced
Cortellini et al. [28]	2021	65.7	70	NSCLC	First	Advanced
Ochi et al. [29]	2021	79	69	NSCLC	All	Advanced
Rounis et al. [30]	2021	83.3	69	NSCLC	Second	Advanced
Nyein et al. [31]	2022	53	65	NSCLC	All	Advanced
Ng et al. [32]	2024	85.7	69	HCC	All	Advanced
Metselaar-Albers et al. [33]	2024	44	65	MM; NSCLC	First	Advanced
Wang et al. [34]	2024	78.1	60	HCC; CRC; Gastric Cancer	All	Advanced
Rousseau et al. [35]	2025	67	65	NSCLC	First	Advanced

* The data refer to two independent cohorts, for which outcomes are reported within the same study. CRC: Colorectal Cancer; GC: Gastric Cancer; GIST: GastroIntestinal Stromal Tumor; HCC: Hepatocellular Carcinoma; MM: Malignant Melanoma; NR: Not Reported; NSCLC: Non-Small Cell Lung Cancer; RCC: Renal Cell Carcinoma; SCLC: Small Cell Lung Cancer; UC: Urothelial Carcinoma.

**Table 3:** Temporal and qualitative characteristics of ABT across the included studies.

Author	Year	ABT Use Window	Type of ABT *
Sen et al. [21]	2018	Within 30–60 days prior to ICI initiation or during ICI therapy	Quinolones; beta-lactams; tetracyclines
Derosa et al. ** [22]	2018	Within 30–60 days prior to ICI initiation	Beta-lactams; quinolones; tetracyclines; aminoglycosides
Derosa et al. ** [22]	2018	Within 30–60 days prior to ICI initiation	Beta-lactams; quinolones; tetracyclines; macrolides; sulfonamides; nitromidazole
Pinato et al. [23]	2019	Within 30 days prior to ICI initiation or concurrent with ICI therapy	Beta-lactams; quinolones; tetracyclines; macrolides; sulfonamides; nitromidazole; aminoglycosides
Schett et al. [24]	2019	From 2 months prior to ICI initiation to 1 month after ICI discontinuation	Beta-lactams; chinolone; macrolide
Hopkins et al. (IMVigor210) ** [25]	2020	From 30 days prior to 30 days after ICI initiation	NR
Hopkins et al. (IMVigor211) ** [25]	2020	From 30 days prior to 30 days after ICI initiation	NR
Chalabi et al. [26]	2020	From 30 days prior to 30 days after ICI initiation	Quinolones; penicillins; cephalosporin; macrolide; carbapenem; glycopeptide; lincomycin; oxazolidinone
Guven et al. [27]	2021	From 3 months prior to 3 months after ICI initiation	Quinolones; amoxicillin-clavulanic acid; clarithromycin and metronidazole; piperacillin-tazobactam; ceftriaxone
Cortellini et al. [28]	2021	Within 30 days prior to ICI initiation	NR
Ochi et al. [29]	2021	From 2 months prior to 1 month after ICI initiation	Beta-lactams; carbapenem; new quinolone; macrolide; tetracycline; trimethoprim-sulfamethoxazole; others
Rounis et al. [30]	2021	Within 30 days prior to ICI initiation or during ICI therapy (first 12 weeks)	NR
Nyein et al. [31]	2022	Within 60 days prior to ICI initiation or concurrent with the first month of ICI therapy	Levofloxacin; cefazolin; azithromycin
Ng et al. [32]	2024	Within 30 days prior to or during ICI therapy	NR
Metselaar-Albers et al. [33]	2024	Up to >365 days prior to ICI initiation	NR
Wang et al. [34]	2024	From 30 days prior to ICI initiation until ICI discontinuation	NR
Rousseau et al. [35]	2025	From 60 days to 42 days prior to ICI initiation	Fluoroquinolone; macrolide; sulfonamide; pennICIsllin; other beta-lactams

* ranked by frequency. ** The data refer to two independent cohorts, for which outcomes are reported within the same study. ABT: systemic AntiBiotic Therapy; ICIS: Immune Checkpoint Inhibitor; NR: Not Reported.

## Data Availability

No new data were created or analyzed in this study. Data sharing is not applicable to this article.

## Supplementary Materials

The following supporting information can be downloaded at: https://www.mdpi.com/article/10.3390/cancers18050869/s1, Figure S1: Flowchart for studies’ selection; Table S1: Quality assessment of included studies; File S1: PRISMA 2020 Checklist; Figure S2a,b: Separate pooled analysis of OS data in patients treated with ICIs in combination with chemotherapy; File S2: Temporal eligibility criteria and hazard ratio selection; File S3: HR for OS and PFS. File S4: Sensitivity analysis in NSCLC.
