# Supplementary material for: A Silent Saboteur of Immunotherapy: Antibiotic Use and Its Impact on Immune Checkpoint Inhibitors Efficacy, a Systematic Review and Meta-Analysis of Recent Studies
Source: Cancers (Basel). 2026 Mar 8;18(5):869. doi: 10.3390/cancers18050869 (PMC12984459; doi:10.3390/cancers18050869)
Supplement: Supplementary file 1 [file cancers-18-00869-s001.zip › Supplementary File S2.pdf]

Supplementary File S2

Temporal eligibility criteria and hazard ratio selection

After thorough discussion and consensus among the authors, we decided a priori to include studies published from 2018 onwards that evaluated patients treated with immune checkpoint inhibitors (ICIs) and explicitly reported the temporal window of antibiotic therapy (ABT) exposure. This temporal restriction was justified by the rapid evolution and widespread clinical adoption of modern ICIs since 2018, ensuring that included studies reflect contemporary treatment protocols, dosing regimens, and patient management strategies.

A key and innovative aspect of our meta-analysis was the a priori selection of the hazard ratio (HR) corresponding to the ABT exposure window closest to ICI administration. In studies reporting multiple temporal windows—for instance, including ABT exposure up to 365 days before ICI initiation—we consistently selected the HR corresponding to ABT use during ICI therapy. Studies that did not explicitly report the ABT temporal window or that analyzed patients receiving ABT too distant from ICI administration were excluded. This approach strengthens the biological plausibility of our findings, as ABT exposure contemporaneous with immunotherapy is most likely to influence the host immune response and treatment outcomes.

These a priori decisions reduce heterogeneity and ensure that our pooled estimates are both clinically interpretable and mechanistically meaningful. By focusing on ABT exposure proximal to ICI administration, our meta-analysis provides an updated, biologically grounded synthesis of the potential impact of ABT on immunotherapy efficacy, while minimizing confounding from more distant exposures.
