# Supplementary material for: A Silent Saboteur of Immunotherapy: Antibiotic Use and Its Impact on Immune Checkpoint Inhibitors Efficacy, a Systematic Review and Meta-Analysis of Recent Studies
Source: Cancers (Basel). 2026 Mar 8;18(5):869. doi: 10.3390/cancers18050869 (PMC12984459; doi:10.3390/cancers18050869)
Supplement: Supplementary file 1 [file cancers-18-00869-s001.zip › Supplementary File S3.pdf]

Supplementary File S3.

Summary of hazard ratios (HR) for overall survival (OS) and progression-free survival (PFS)

Author	Year of Publication	HR (OS)	95% CI (OS)	HR (PFS)	95% CI (PFS)
Sen [21]	2018	2.0	1.2–3.3	0.7	0.4–1.3
Derosa* [22]	2018	1.9	0.8–4.7	2.3	1.2–4.4
Derosa* [22]	2018	2.0	1.3–3.2	1.2	0.9–1.7
Pinato [23]	2019	0.9	0.5–1.4	NR	NR
Schett [24]	2019	1.1	0.75–1.63	0.86	0.61–1.22
Hopkins* [25]	2020	1.44	1.19–1.73	1.24	1.05–1.46
Hopkins* [25]	2020	0.95	0.71–1.25	1.24	1.05–1.46
Chalabi [26]	2020	1.32	1.06–1.63	1.17	0.97–1.4
Guven [27]	2021	2.306	1.155–4.601	2.238	1.284–3.9
Cortellini [28]	2021	1.47	1.17–1.84	1.31	1.06–1.62
Ochi [29]	2021	1.38	1.033–1.844	1.141	0.892–1.46
Rounis [30]	2021	1.35	0.761–2.406	1.655	0.068–2.83
Nyein [31]	2022	1.35	0.91–2.02	NR	NR
Ng [32]	2024	1.67	0.8–3.49	1.09	0.53–2.25
Metselaar-Albers [33]	2024	1.03	0.81–1.3	NR	NR
Wang [34]	2024	0.8	0.6–1.1	0.7	0.6–0.9
Rousseau [35]	2025	1.02	0.97–1.08	NR	NR

NR: Not Reported; *The data refer to two independent cohorts, for which outcomes are reported within the same study.
