# Supplementary material for: A Silent Saboteur of Immunotherapy: Antibiotic Use and Its Impact on Immune Checkpoint Inhibitors Efficacy, a Systematic Review and Meta-Analysis of Recent Studies
Source: Cancers (Basel). 2026 Mar 8;18(5):869. doi: 10.3390/cancers18050869 (PMC12984459; doi:10.3390/cancers18050869)
Supplement: Supplementary file 1 [file cancers-18-00869-s001.zip › Supplementary File S4.pdf]

Supplementary File S4

Sensitivity analysis in NSCLC

A sensitivity analysis was conducted restricting the meta-analysis to patients with non–small cell lung cancer (NSCLC), representing the largest and most methodologically homogeneous subgroup among the included studies [21-24,26,28-31,33,35]. This analysis was performed to assess the robustness of the association between systemic antibiotic therapy (ABT) exposure and survival outcomes in a single tumor type supported by an adequate sample size.

For overall survival (OS), a total of 45,896 NSCLC patients were included. Statistical heterogeneity was moderate and statistically significant (Q = 14.28, df = 7, P = 0.046), with an I^2 of 50.98%, indicating relevant between-study variability. The pooled fixed-effect analysis yielded a hazard ratio (HR) for OS of 1.05 (95% CI: 0.996–1.11). This result suggests a trend toward worse overall survival in patients receiving antibiotics compared with those not exposed; however, the confidence interval narrowly crossed unity, indicating that the association did not reach formal statistical significance.

For progression-free survival (PFS), the same cohort of NSCLC patients was analyzed. In contrast to OS, no statistically significant heterogeneity was observed (Q = 4.29, df = 5, P = 0.51; I^2 = 0%), supporting a high degree of consistency across studies. The pooled HR for PFS was 1.16 (95% CI: 1.02–1.30), with identical estimates obtained under both fixed- and random-effects models. This finding indicates a statistically significant increase in the risk of disease progression associated with ABT exposure in NSCLC patients treated with ICIs.
