# Supplementary material for: A Silent Saboteur of Immunotherapy: Antibiotic Use and Its Impact on Immune Checkpoint Inhibitors Efficacy, a Systematic Review and Meta-Analysis of Recent Studies
Source: Cancers (Basel). 2026 Mar 8;18(5):869. doi: 10.3390/cancers18050869 (PMC12984459; doi:10.3390/cancers18050869)
Supplement: Supplementary file 1 [file cancers-18-00869-s001.zip › Supplementary Table S1.pdf]

Supplementary Table S1. Quality assessment of included studies

First author	Year	Study Design	MINORS score	NOS score
Sen et al. [21]	2018	Post hoc analysis	12	5
Derosa et al. [22]	2018	R	15	7
Pinato et al. [23]	2019	P	17	8
Schett et al. [24]	2019	R	16	7
Hopkins et al. [25]	2020	Post hoc analysis	17	8
Chalabi et al. [26]	2020	R	15	8
Guven et al. [27]	2021	R	16	6
Cortellini et al. [28]	2021	P	16	9
Ochi et al. [29]	2021	R	16	7
Rounis et al. [30]	2021	P	18	8
Nyein et al. [31]	2022	R	15	7
Ng et al. [32]	2024	R	18	8
Metselaar-Albers et al. [33]	2024	R	19	9
Wang et al. [34]	2024	R	17	9
Rousseau et al. [35]	2025	R	20	9

MINORS: Methodological Items for Non-randomized Studies; NOS: Newcastle - Ottawa quality assessment Scale; P: Prospective; R: Retrospective.
