# Supplementary figures and images for: A Silent Saboteur of Immunotherapy: Antibiotic Use and Its Impact on Immune Checkpoint Inhibitors Efficacy, a Systematic Review and Meta-Analysis of Recent Studies
Source: Cancers (Basel). 2026 Mar 8;18(5):869. doi: 10.3390/cancers18050869 (PMC12984459; doi:10.3390/cancers18050869)

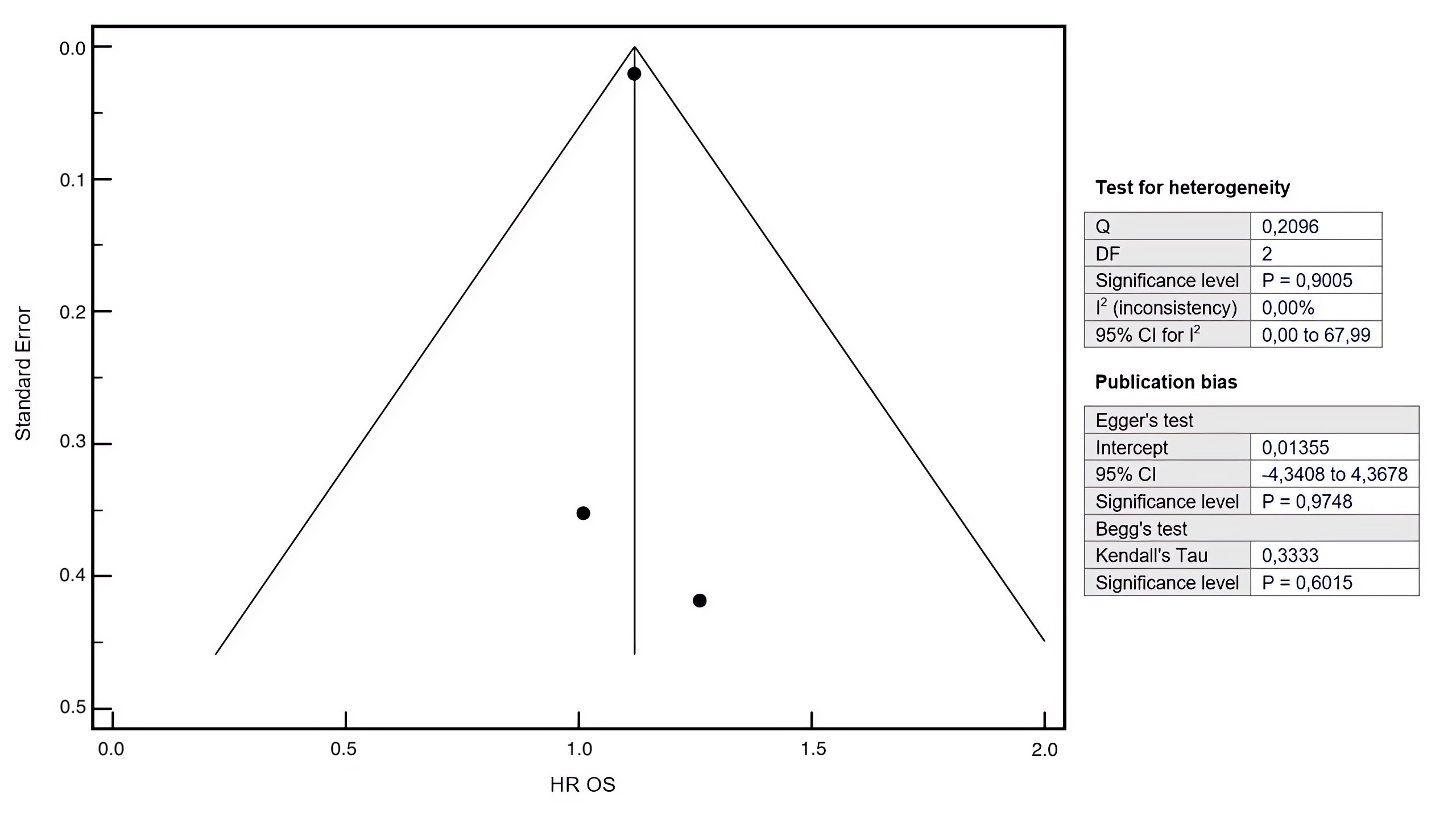

Supplement: Supplementary file 1 [file cancers-18-00869-s001.zip › Supplementary Figure S2a.jpg]

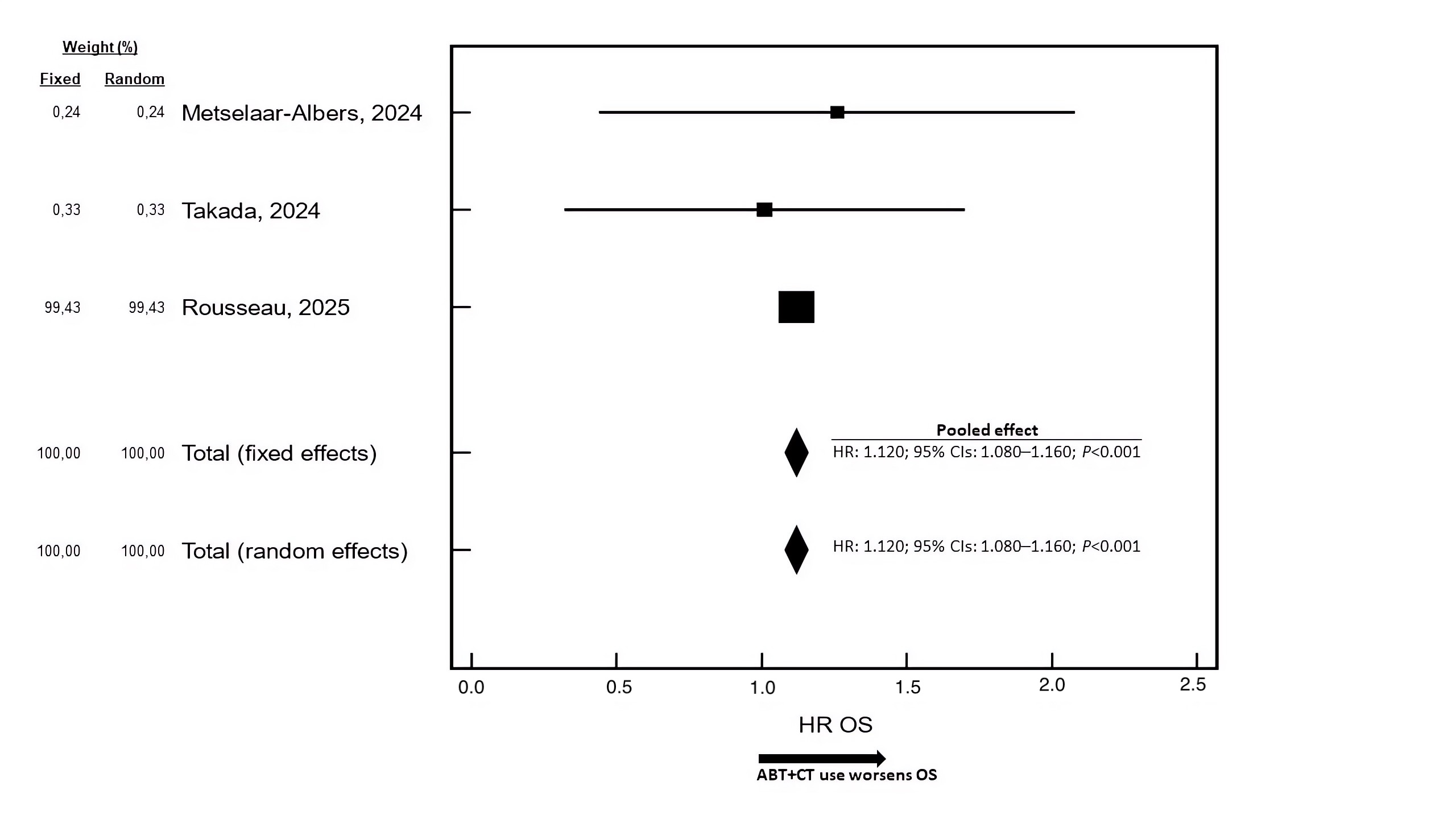

Supplement: Supplementary file 1 [file cancers-18-00869-s001.zip › Supplementary Figure S2b.jpg]

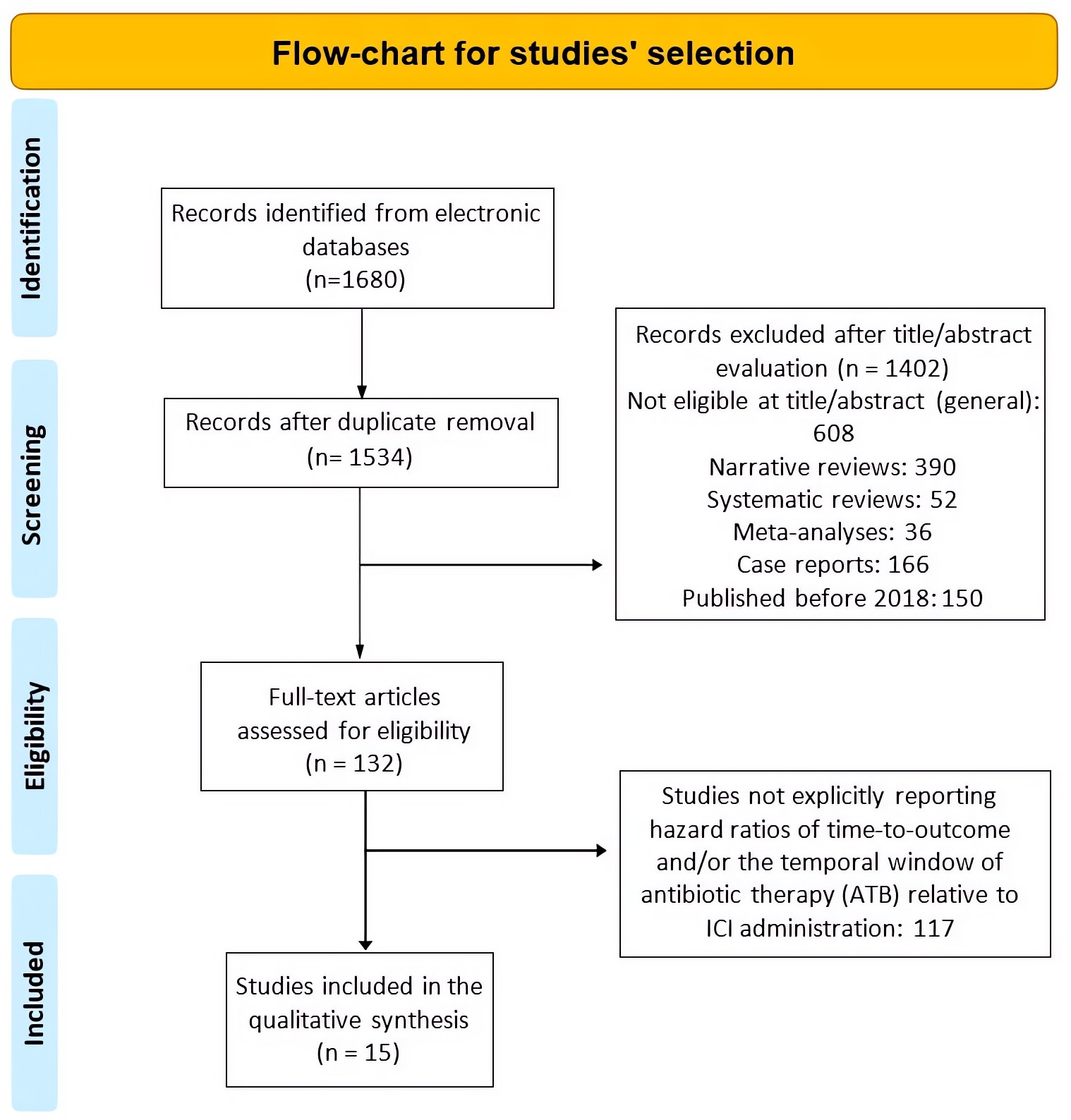

Supplement: Supplementary file 1 [file cancers-18-00869-s001.zip › Supplementaty Figure S1.jpg]
